# Increasing the Realism of *in Silico* pHLIP Peptide Models with a Novel pH Gradient CpHMD Method

**DOI:** 10.1021/acs.jctc.2c00880

**Published:** 2022-10-18

**Authors:** Tomás F. D. Silva, Diogo Vila-Viçosa, Miguel Machuqueiro

**Affiliations:** BioISI − Instituto de Biosistemas e Ciências Integrativas, Faculdade de Ciências, Universidade de Lisboa, 1749-016Lisboa, Portugal

## Abstract

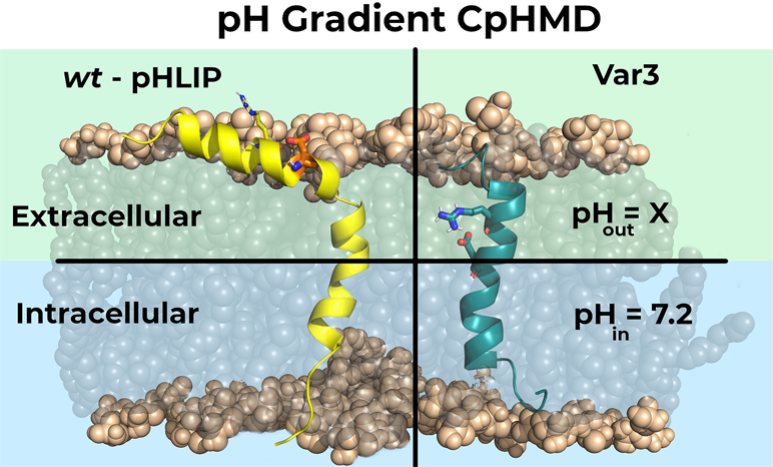

The pH-low insertion peptides (pHLIP) are pH-dependent membrane inserting peptides, whose function depends on the cell microenvironment acidity. Several peptide variants have been designed to improve upon the *wt*-sequence, particularly the state transition kinetics and the selectivity for tumor pH. The variant 3 (Var3) peptide is a 27 residue long peptide, with a key titrating residue (Asp-13) that, despite showing a modest performance in liposomes (p*K*^ins^ ∼ 5.0), excelled in tumor cell experiments. To help rationalize these results, we focused on the pH gradient in the cell membrane, which is one of the crucial properties that are not present in liposomes. We extended our CpHMD-L method and its pH replica-exchange (pHRE) implementation to include a pH gradient and mimic the pHLIP-membrane microenvironment in a cell where the internal pH is fixed (pH 7.2) and the external pH is allowed to change. We showed that, by properly modeling the pH-gradient, we can correctly predict the experimentally observed loss and gain of performance in tumor cells experiments by the *wt* and Var3 sequences, respectively. In sum, the pH gradient implementation allowed for more accurate and realistic p*K*_a_ estimations and was a pivotal step in bridging the *in silico* data and the *in vivo* cell experiments.

## 1 Introduction

The pH-low insertion peptides (pHLIP) constitute a family of long transmembrane peptides (∼27–36 amino acid residues), whose function and folding depend on the extra-cellular acidity to adopt a membrane-inserted state.^1−6^ An α-helical fold, usually adopted at pH values below ∼6.0,^3,7^ is characterized by a transmembrane configuration (State III) with a kink in the water–membrane region.^7^ At more basic conditions, pHLIP peptides typically unfold to a random coil conformation, either in solution (pH > 8.0) (State I) or adsorbed to the membrane surface (pH 7.0 to 8.0) (State II).^2^ Although the folding process has been challenging to describe, as shown in recent work regarding the insertion transition states,^8^ the characteristic pH dependency relates to key titrating residues identified as regulators of this insertion process, such as Asp-14 in the *wt* peptide, and their local interactions with the surrounding environment. Indeed, during the design process of several variant sequences, an aspartate residue found in a key location was essential.^4^ In equilibrium, this residue accesses the water–membrane interface region, at least transiently, and acts as a pH probe, sensing when pH is high enough to trigger deprotonation and membrane exiting.

The pHLIP *wt* peptide showed promising targeting features for tumors, inflammation tissues, and ischemia^5,9^ or as a model peptide to study the molecular interactions regulating transmembrane peptide thermodynamic stability.^7,10−12^ However, the lack of tumor specificity and slow kinetics of insertion, in *in vivo* studies, prevented widespread therapeutic use of *wt* pHLIP.^6^ To overcome these limitations, the Andreev Lab designed a systematic study with 16 pHLIP peptide variants to get a thorough assessment of their liposome membrane partitioning, peptide stability, and membrane insertion p*K* values (p*K*^ins^).^4^ Subsequent *in vivo* studies also measured the tumor targeting ability and organ distribution of some of these variants.^13^ Among all tested peptides, Variant 3 (Var3) was a distinct case that showed poorer performance than the *wt* peptide in liposome models. However, *in vivo* studies showed that it excelled in coupling faster kinetics with an improved tumor/kidney target ratio.^4,13,14^ The Var3 peptide is a 27 amino-acid long peptide with a key aspartate (Asp-13), a shorter α-helix, and fewer C-terminus titrating acidic residues. This reduced number of anionic residues improves the kinetics rate of membrane insertions by facilitating their transient protonation. Furthermore, the smaller p*K*^ins^ (5.0), measured in liposomes,^4^ suggests that the tumor-targeting ability is impaired as only a small fraction of the peptide accumulates in tumor cells (pH ∼ 6.0–6.8). Nevertheless, fluorescence imaging data showed an improved therapeutic index relative to the *wt*,^15^ reduced liver accumulation, faster organ clearance,^14^ the wider time window for imaging, and the best tumor/organ ratio of all variants.^4^ In sum, this data strongly indicates a higher p*K*^ins^*in vivo* than the one observed for liposomes.

The pHLIP therapeutic performance depends on a good match between the p*K*^ins^ value and the tumor microenvironment (TME) acidity.^16,17^ Yet, the aforementioned results highlight a fundamental flaw in extrapolating liposome p*K*^ins^ to cells and *in vivo* experiments. Indeed, promising peptide sequences may have been discarded on the account of poor liposomal performance or the p*K*^ins^ falling outside the optimum pH region. Nevertheless, liposomal studies are quite standard and provide essential data to validate *in silico* results, which, as expected, use simple membrane models.^7,12,18^

*In silico* studies of pHLIP peptides successfully provided novel insights, with molecular-level detail, into pHLIP function and structure, prompting the rational design of fine-tuned variants. Several studies focused on the structural stability,^18,19^ (de-)insertion kinetics, possible metastable states, and electrostatic interactions modulating the pH dependency.^7,12,20,21^ Most of them used 2-oleoyl-1-palmitoyl-*sn*-glycero-3-phosphocholine (POPC) membrane bilayers to mimic liposomal conditions, such as ionic strength, pH, and peptide state.^18,19,22^ Particularly, constant-pH molecular dynamics (CpHMD) simulations are helpful, as the residues are titrating at specific pH values, allowing the conformational and protonation sampling to be coupled. These methods may treat the protonation either as discrete^7,12,23−45^ or continuous.^46−59^ Consequently, they promote the study of complex peptide–membrane configurations and the impact of the key titrating residues positioning along the membrane normal on helical (un)folding and side-chain interactions. The electrostatic environment around the key aspartate residues changes with the peptide movement, favoring either insertion or exiting processes through protonation or deprotonation events, respectively. Additionally, the transient protonation of C-terminus anionic residues seems to play a major role in the associated kinetics of transition between the inserted and adsorbed states.^3,7^ Our previous work focused on describing the electrostatic network dictating the pHLIP–membrane thermodynamic equilibrium in state III of *wt* and its L16H peptide variant.^7^ Furthermore, we also improved the peptide–membrane configuration sampling by coupling a replica-exchange scheme to the CpHMD methodology (pHRE method).^12^ We validated our implementation by comparing multiple p*K*_a_ profile calculations of Asp-14, in differently sized membrane patches, with the experimental p*K*^ins^ ∼ 6.0.

The computational models we developed are state-of-the-art in the study of pH-sensitive peptides interacting with lipid bilayers. Nonetheless, there are still several avenues that can, and should, improve the realism of the process. These include the use of more representative lipid mixtures in the bilayer, incorporating a membrane electrochemical potential, or the correct modeling of a transmembrane pH gradient. In particular, our model should mimic more closely crucial cell features that are usually modified in tumors. In particular, the transmembrane pH gradient, that exists between the intracellular and extracellular compartments,^60,61^ has an increased role in tumors, due to the TME formation.^16^ Furthermore, this gradient is absent in liposomes, affecting pHLIP–membrane equilibrium, p*K*_a_ shifts in key residues, and ultimately, the accuracy of our predictive model when comparing liposomes and cell experiments. The development of a transmembrane pH gradient method within pHRE is, to the best of our knowledge, the first effort to perform pH gradient simulations of transmembrane peptides. This newly developed protocol seeks to mimic *in situ* conditions to improve our pHLIP–membrane model accuracy and robustness while helping bridge the gap between *in silico* models, liposomes, and living cells.

We successfully implemented the pH gradient within our pHRE methodology and significantly improved our model of the local residue interactions that define peptide performance in cellular environments. We also evaluated how this more realistic description may impact our model’s accuracy and predictive ability. With this goal, we calculated p*K*_a_ profiles, in gradient and nongradient conditions, and performed a detailed quantification of the interactions between the key aspartate and the neighboring electrostatic partners along the membrane normal. This systematic analysis of *wt* and Var3 peptide variants under a pH gradient setup proved pivotal in improving our knowledge of the local changes in the interaction networks that propelled their distinct *in vivo* performances.

## 2 Methods

### 2.1 System Setup and pH-Gradient Implementation

**Figure 1:**
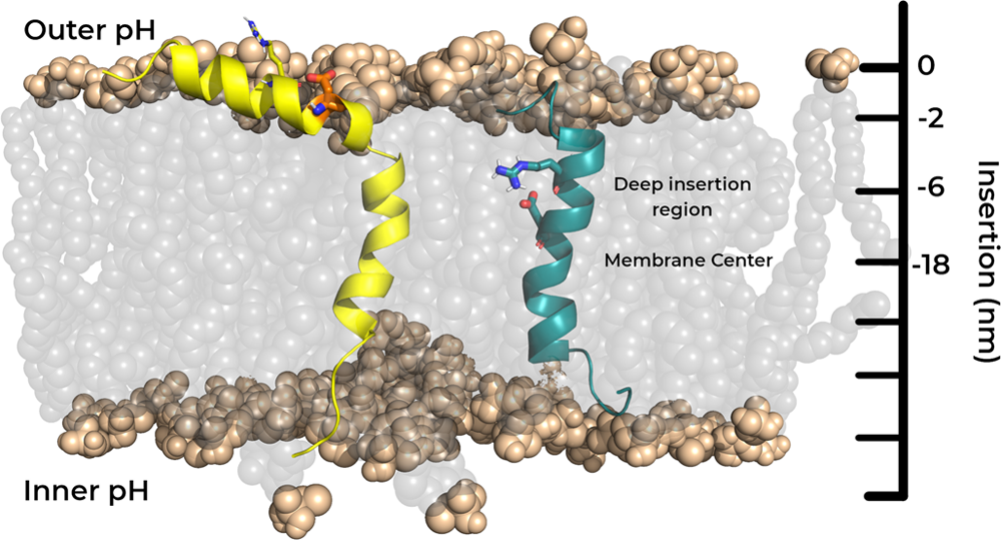
Graphical representation of *wt* (left) and Var3 (right) peptides inserted in a POPC bilayer. Although the two peptides are illustrated together, in the same lipid bilayer, they are modeled in separate membrane patches. The *wt* and Var3 peptides are represented in yellow and teal, respectively, while the phosphate groups (both the P and the O atoms) of the membrane are represented as light brown spheres. The key pH sensor residues, Asp-14 (*wt*) and Asp-13 (Var3), and the two important arginine residues, Arg-11 (*wt*) and Arg-9 (Var3), are also highlighted as sticks.

For this work, two distinct systems were simulated using a pre-equilibrated membrane–pHLIP structure composed of the *wt* (ACEQNPIYWA**R**YA**D**WLFTTPLLLLDLALLVDADEGT)^2^ or the Var3 (ACDDQNPW**R**AYL**D**LLFPTDTLLLDLLW) sequences^4^ across a 256 (*wt*) or a 160 (Var3) membrane bilayer of 2-oleoyl-1-plamitoyl-*sn*-glycero-3-phosphocholine (POPC) molecules, respectively. For the *wt* system, the peptide was already placed in a kinked α-helical fold, creating two segments above the 15th and below the 18th residues.^7^ Meanwhile, the Var3 peptide started in a full helix structure and, throughout the equilibration protocol, it converged to a similar structural conformation (Figure 1). For both the *wt* and the Var3 systems, the peptide was allowed to equilibrate through a 2-fold optimization protocol: first, a molecular dynamics (MD) simulation using position restraints (1000 kJ/mol nm^2^) on the peptide to allow the lipids to accommodate the peptide conformation; second, an unrestrained CpHMD simulation, at pH 6.0, to equilibrate both the conformation and the protonation states of the titrating residues.

pHRE is an enhanced sampling method extended from the CpHMD-L methodology,^23,25,34−36^ which can be described in three modules: a Poisson–Boltzmann/Monte Carlo (PB/MC) step from which new protonation states are generated for titrable groups using PB-derived free energy terms; a solvent relaxation step where solvent molecules (SPC water model) are allowed to adapt to the new protonation states; and a final molecular mechanics/molecular dynamics (MM/MD) production step where new conformations are sampled. The Baptista’s PB/MC implementation of the pH gradient^61^ was integrated into our pHRE methodology. In this setup, the membrane center defines the border between the two regions with different pH values. Prior to the simulation, an outer pH (pH_out_) is assigned to every residue whose starting Z position is above the membrane center, while residues below the membrane center are assigned to an inner pH (pH_in_).

To perform nongradient pHRE simulations, *n* simultaneous CpHMD-L simulations, known as pH replicas, are running, each with an assigned pH value. During the MM/MD procedure, the replicas are stopped to allow a pH exchange attempt between adjacent values, with a fixed frequency (*tau*_*RE*_). When using the pHRE gradient setup, only the external (pH_out_) is chosen from the set of *n* pH values, since pH_in_ is fixed at the physiological value (7.2). Therefore, only pH_out_ is allowed to exchange between replicas. If a pH replica-exchange move is accepted, according to the probability given by eq 1, conformation and protonation are swapped between different pH values, increasing the variability of our sampling at low and high energy states for every replica.
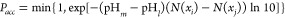
1pH_m_ and pH_l_ are the exchanging pH values, *N*(*x*_*i*_) and *N*(*x*_*j*_) are the number of protonated groups for the *x*_*i*_ and *x*_*j*_ states. Ten replicates of 100 ns each were simulated for both peptide systems in the gradient and nongradient setups, running a total of 4 system combinations: *wt* gradient and *wt* nongradient and var3 gradient and var3 nongradient. The initial 50 ns of each replicate were discarded to ensure a good equilibration of the structural properties. Each replicate consisted of four pH replicas, each value given from the range of 4.0 to 7.0 with a step of 1.0. Each CpHMD cycle was 20 ps (*tau*_*prot*_), with a relaxation step of 0.2 ps (*tau*_*rlx*_), and the pH exchanges were attempted at every 20 ps (*tau*_*RE*_) out of phase from *tau*_*prot*_.^62^

The titrating residues were the (N- and C-) termini: *wt*, Asp-14, 25, 31, 33, and Glu-34; Var3, Cys-2, Asp-3, 4, 13, 19, 24, and 27. The peptide starting conformations, for each replicate, were obtained from the final segment of the previously mentioned equilibration protocol. These structures were extracted from the final part of the equilibration step with at least 1 ns difference between each other, to ensure that they were thermodynamically stable and not too structurally correlated. The *wt* peptide showed the typical conformation with a partial loss of helical content around the residues 15–18, as previously observed.^7^ However, Var3 showed a significantly different peptide conformation, probably due to its different size and amino acid sequence (Figure 1).

### 2.2 Molecular Dynamics Settings

Every system was simulated using a modified version^63,64^ of the GROMACS 4.0.7 package,^65^ the GROMOS 54A7 force field,^66^ and a Python-based wrapper to apply the pH replica-exchange method.^12,62^ The nonbonded interactions treatment was done with a single-cutoff scheme updating the forces, at every step, for all pairs below a 14 Å cutoff.^37^ Regarding long-range interactions, the van der Waals interactions were truncated at 14 Å, while the electrostatic interactions were treated with a generalized reaction field (GRF) method, using a dielectric constant of 54^67^ and ionic strength of 0.1 M. Lipid and peptide bond lengths were constrained using the P-LINCS algorithm,^68^ and water molecules are treated as simple point charges (SPCs),^69^ using the SETTLE algorithm.^70^ In MD simulations, the integrator time step used was 2 fs and conformations were generated from an NPT ensemble. The v-rescale temperature bath,^71^ at 310 K, was coupled separately to the solute (peptide and membrane) and solvent with a relaxation time of 0.1 ps. The system pressure was kept constant at 1 bar with a Parrinello–Rahman barostat, with a relaxation time of 5 ps and compressibility of 4.5 × 10^–5^ bar^–1^.

### 2.3 Poisson–Boltzmann/Monte Carlo Simulations

Poisson–Boltzmann calculations were performed with the Delphi V5.1 program^72^ using both partial charges and Lennard-Jones parameters of the GROMOS 54A7 force field to derive the atom radii at 2 RT.^73^ The peptide–membrane molecular surface was described using a 1.4 Å radius probe, an ion-exclusion layer of 2.0 Å, and ionic strength of 0.1 M. The dielectric was set as 2 and 80, for solute and solvent, respectively. The conducted two-step focusing procedure employed two 91 point grids, where the coarse grid had an ∼1 Å spacing between grid points, and the smaller grid had a spacing of ∼0.25 Å. In the coarse grid, the relaxation parameters were 0.20 and 0.75 for linear and nonlinear iterations, respectively, while periodic boundary conditions were applied in the *xy* plane. Background interaction calculations were truncated at 25 Å, and the electrostatic potential convergence threshold was 0.01 kT/e.

Monte Carlo (MC) calculations sample the protonation states of all titrating residues using a modified version of the PETIT program that implements the pH gradient setup, as explained in the cited protocol.^61^ In this new version, the program requires a preassignment of each titrating site to one of two proton baths, each with a distinct pH value, representing the inner monolayer (pH_*in*_) or the outer (pH_*out*_) monolayer.^60,61^ The site assignment was static and required a previous insertion analysis, for each titrating residue using the unrestrained CpHMD equilibration data, to determine which monolayer was being populated on average to choose the correct proton bath. The analysis was done for both peptides, where Asp-25 (*wt*) and Asp-19 (Var3) assignment needed to be more cautious since their close position to the membrane center (average position of all P atoms in the membrane normal) allows crossing between both monolayers. Due to previous data on the role of the membrane center aspartate^7^ and their average positions, both residues were assigned to the outer monolayer, where Asp-25 and Asp-19 may impact the membrane insertion of Asp-14 and Asp-13, respectively. Nevertheless, their central membrane position prevents the deprotonation of the two residues; hence, their monolayer assignment has no impact in the final results. In the CpHMD simulations setup, the pH gradient is defined by two user-specified parameters, the pH and the ΔpH. These are used to calculate pH_*in*_ and pH_*out*_ from pH ± (ΔpH/2).^61^ Regarding the non pH gradient simulations, all titrating residues are exposed to a single proton bath at a given pH value in the previously established pH range. The PB protonation energy terms and the assigned pH of the residue (pH gradient) or the solution pH (non pH gradient) are used in a Metropolis scheme to calculate the probability of protonation change. Proton tautomerism was taken into account for all titrable groups. For each conformation, 10^5^ MC cycles were performed, where each cycle corresponds to a trial change of each individual site and pairs of sites with an interaction larger than 2 p*K* units.

### 2.4 Analyses and Error Calculations

In this work, Asp-13 (Var3) and Asp-14 (*wt*) protonation is the major membrane insertion trigger. These events are modulated by the phosphate interaction shell and other relevant properties, affected by the peptide–membrane configuration, that require specialized calculations.

The equilibration of membrane-related properties, such as bilayer thickness and residue membrane insertion, in peptide–membrane complexes, is very convoluted due to the local deformation induced by the peptide. The standard bilayer thickness calculations use the arithmetic difference of the average Z positions of both monolayer lipids; hence, affected lipids dilute the bilayer thickness values and prevent a fair assessment of the membrane health. To overcome this issue, our equilibration analyses focused on two membrane regions: the local deformation and the “bulk” unaffected lipids—lipid distance to the peptide > 15 Å. In our approach, we quantify the local deformation and discriminate these two regions by calculating the half thickness values, for each monolayer, of an annulus region—defined by two radii centered on the peptide. An annulus scan on the *xy* plane describes the membrane monolayer outline which, at longer distances, should converge to the experimental POPC half thickness range. All equilibrated conformation snapshots are considered in the calculations and the experimental POPC half thickness range was obtained by interpolating from experimental bilayer thickness measurements in the fluid range at different temperatures.^74^ The membrane local deformation also presented in this work was calculated as the difference between the local half thickness and the half thickness of the bulk region (beyond the 15 Å cutoff).

To obtain the necessary residue membrane insertion data, analyses are performed by defining the closest membrane monolayer surface as the average *Z* position of the lipid phosphate group (P and O) atoms within a 6 Å radius from the group of interest and then calculating the relative position of the residue to the reference. Additionally, to properly account for membrane deformations, the chosen radius has to be small enough to exclude atoms outside of the perturbation affecting the average estimation. Note that an excessively small radius has the downside of lacking enough atoms to properly characterize the membrane interface. To overcome this issue, our current protocol enforces a minimum of 10 atoms (phosphorus and/or oxygen), within the cutoff radius, to define the average Z coordinate for the membrane surface. Otherwise, the 10 closest atoms to the group of interest, in a two-dimensional (*x*/*y*) plane, will be used regardless of the cutoff distance.

The calculated insertion time series is then coupled to the corresponding residue protonation states to obtain p*K*_a_ and protonation profiles, a representation of the residue p*K*_a_ or average protonation values along the membrane normal. These profiles are achieved by assigning the residue protonation values, of each conformation, to an insertion bin according to their respective insertion value. By discriminating the protonation data along insertion windows, it is possible to estimate the residue proton binding affinity (p*K*_a_) or average protonation (at each pH value) at those particular membrane regions. For reproducibility and robustness purposes, all calculations must fulfill a few criteria: (1) each insertion slice (at each pH value and replicate) must have a minimum of 10 conformations of each protonated state; (2) at least three replicates need to contribute for the conformational sampling at each pH value and at least two pH values are required for the fit; and (3) to ensure monotonicity, the average protonation should not fluctuate (by 0.05) above the average of the previous lower pH. If the conditions are met, the average protonations at each pH are calculated, and then they are fitted to the Hill equation to obtain the p*K*_a_ values. For protonation profiles, only the first two criteria are required. The profiling procedure is very useful to ascertain the influence of the changing electrostatic environment on protonation by measuring other residue properties along the membrane depth. Therefore, the procedure was also applied to distances between the residues of interest and neighboring electrostatic partners, such as phosphate groups, water molecules, and other residues. The GROMACS tool package was used to calculate these distances. All analyses were done using in-house software (http://mms.rd.ciencias.ulisboa.pt/#software) and the GROMACS package.

To circumvent fitting issues, all p*K*_a_ error values were estimated with a Bayesian bootstrap approach that performed 1000 bootstraps from our average protonation samples, and in each bootstrap, each sample was assigned a random weight. This approach must fulfill the previously applied criteria to obtain the final p*K*_a_ and error values. A simple standard error of the mean method was used to estimate all the other properties’ error values.

## 3 Results and Discussion

### 3.1 Peptide/Membrane Structural Analyses and Equilibration

Liposome studies have proven to be a reliable and affordable technique to evaluate the features of new transmembrane (TM) peptide sequences in the early development stage. However, most liposomes lack the environmental and complex traits that characterize the cell microenvironment, hindering the transferability of the TM peptides’ performance to *in vivo* conditions.^75^ Similarly, *in silico* models also suffer from the same constrictions. Most CpHMD methodologies mimic those liposomes’ proton availability by applying an equal pH value to the inner and outer monolayer environments. However, with the implementation of a pH gradient in pHRE, it is possible to decrease the gap between most liposome- and cell-like conditions. The *wt*-pHLIP peptide has already been successfully studied in a gradient-free environment,^7,12^ where pHRE simulations reproduced the data from experiments performed in liposomes. In a pH gradient setup, the sampling of the peptide–membrane configuration space should be distinct from the previous simulations ensembles, as the sampled protonation space is strongly coupled to the conformation sampling, reflecting on the peptide and key residue properties.

**Figure 2:**
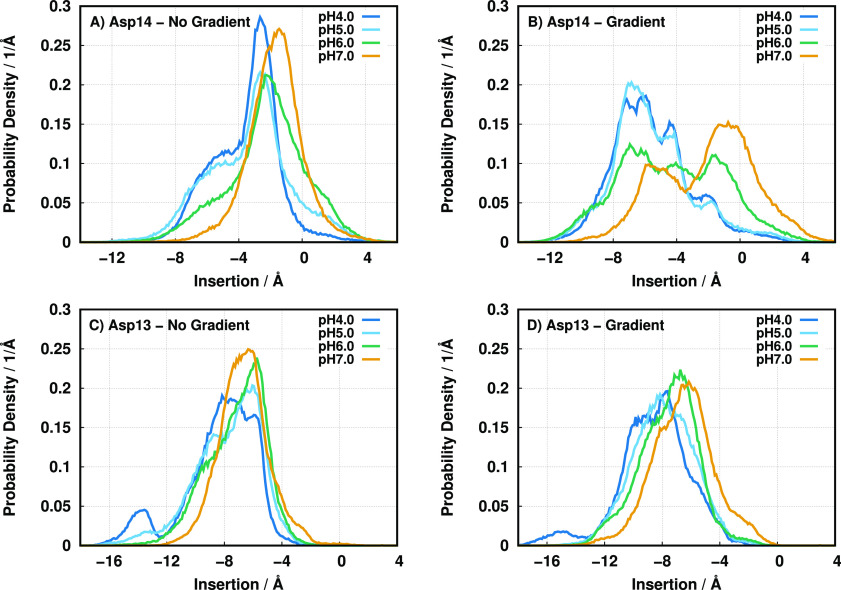
Probability density function of residues Asp-14 of *wt* (A and B) and Asp-13 of Var3 (C and D) populating a given insertion region in a nongradient (A and C) and gradient setup (B and D) at all pH values studied. Negative insertion values correspond to the membrane interior.

The *wt* Asp-14 residue membrane insertion positions clearly highlight the pH gradient method by propelling Asp-14 to sample more abundantly the deeper membrane regions (−10 to −4 Å) relative to the no-gradient setup (Figure 2A,B).

This difference is noticeable even at pH 7.0, where the pH gradient effect is minimal and the two configurational ensembles should tend to converge. This indicates that the pH replica-exchange protocol is effectively promoting the mixing between conformational ensembles and allowing a slightly better sampling of the deeper regions at this high pH value. The Var3 Asp-13 residue, which is structurally and functionally equivalent to Asp-14 in *wt*, possesses a neighboring arginine residue (Arg-9) that is directly above its location in the α-helix (see Figure 1). The shorter peptide length (27 residues) imparts a different peptide structural disposition in the membrane since the shorter helix does not grant much leeway to span the peptide across the bilayer and promotes a higher level of membrane insertion (Figure 2C,D). For this peptide, the presence of a pH gradient in the membrane seems to have only a small effect on the Asp-13 position in the membrane.

**Figure 3:**
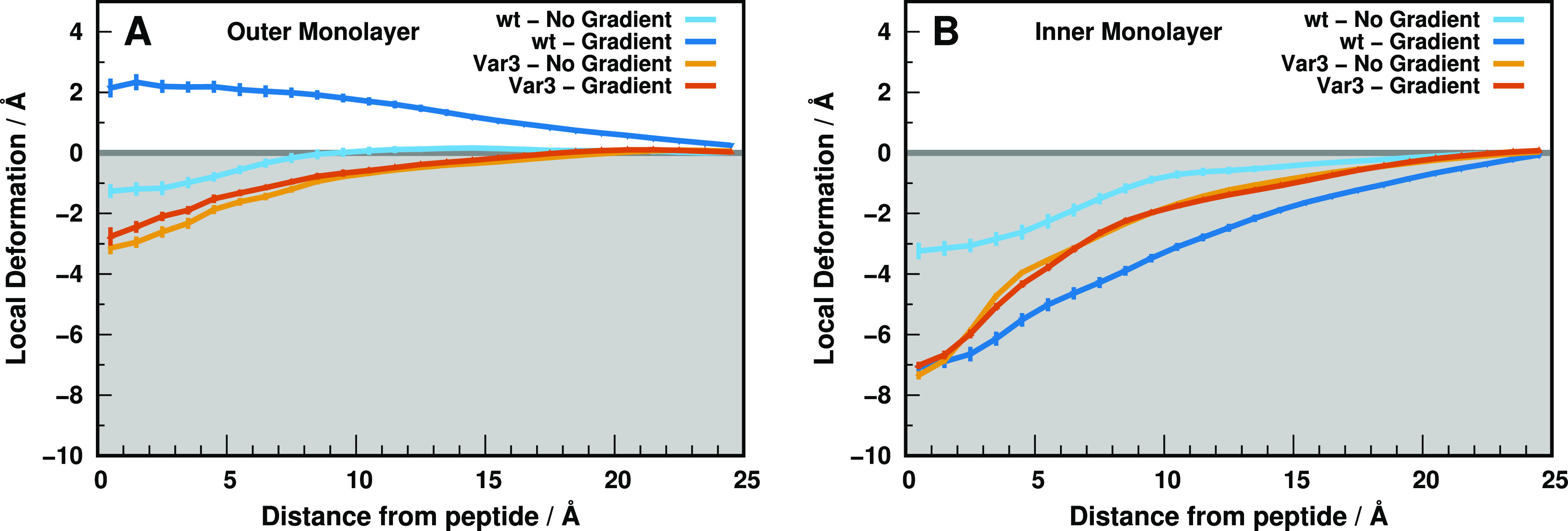
Outer (A) and inner (B) local monolayer deformations induced by the *wt* (blue/cyan) and Var3 (orange/brown) peptides, in both setup conditions at pH 6.0. These deformation values are calculated from the half thickness values calculated along the *xy* plane distance to the peptides (Figure S1 of the Supporting Information). The maximum distance shown (∼25 Å) corresponds to the unperturbed “bulk” lipids. In the replica-exchange scheme, the configurational sampling is shared across all pH replicas; hence, the local deformation profiles at pH 6.0 do not differ significantly from pH 4.0 to 5.0 to 7.0 (see Figures S2–S4 of the Supporting Information). The gray-shaded region corresponds to the membrane interior. The error bars were obtained from all replicates using the standard error of the mean (SEM) and calculated/represented only every 1 Å for clarity.

The distinct membrane behaviors of *wt* Asp-14 may be the result of rearrangements in the peptide-induced local monolayer deformations, structural changes of the peptide itself, or a combination of the two phenomena. Looking at the local membrane deformation (Figure 3), it is evident that both the inner and the outer peptide–membrane configurations are significantly altered by the pH gradient at pH 6.0. In the gradient setup, the higher pH_*in*_ (7.2) induces significant deprotonation of the anionic residues (Asp-31, Asp-33, Glu-34, and C-ter), prompting a larger deformation due to charge repulsion with the phosphate groups coupled with increased water solvation. This membrane invagination of the inner monolayer seems to trigger a small loss of helical content in the peptide segment (Figure S5 of the Supporting Information) and also affects the peptide–membrane equilibration in the outer monolayer. We observe a small protrusion in the outer monolayer (∼2 Å) enveloping the peptide, suggesting an increase in the distance spanned between the two end points of the transmembrane segment, which was confirmed by a small decrease in the peptide helical content (Figure S5 of the Supporting Information). In the Var3 peptide, the gradient setup does not change the already large local membrane perturbation, compared to the nongradient setup (Figure 3). Furthermore, this shorter variant requires more structural unfolding of the α-helix (mainly on the C-terminus) to cross the lipid bilayer (Figure S5 of the Supporting Information). There are small differences between setups, even at pH 7.0 where the gradient effect should have dissipated. This suggests that the Var3 C-terminus region helicity is quite sensitive to the equilibration procedure. However, these different configurational ensembles do not seem to have any major impact on the remaining structural properties around Asp-13 (Figures 2C,D and S1–S6 of the Supporting Information).

Overall, the *wt* peptide in state III seems more thermodynamically unstable in a gradient than in the nongradient setup. These high energy configurations seem to be predominant in the Var3 peptide, independently of the gradient setup used. The membrane deformations observed may alter the electrostatic network around the Asp residues, possibly changing their insertion p*K*_a_ values and respective peptide performance.

### 3.2 Asp-14 and Asp-13 Membrane Insertion p*K*_a_ Profiles

**Figure 4:**
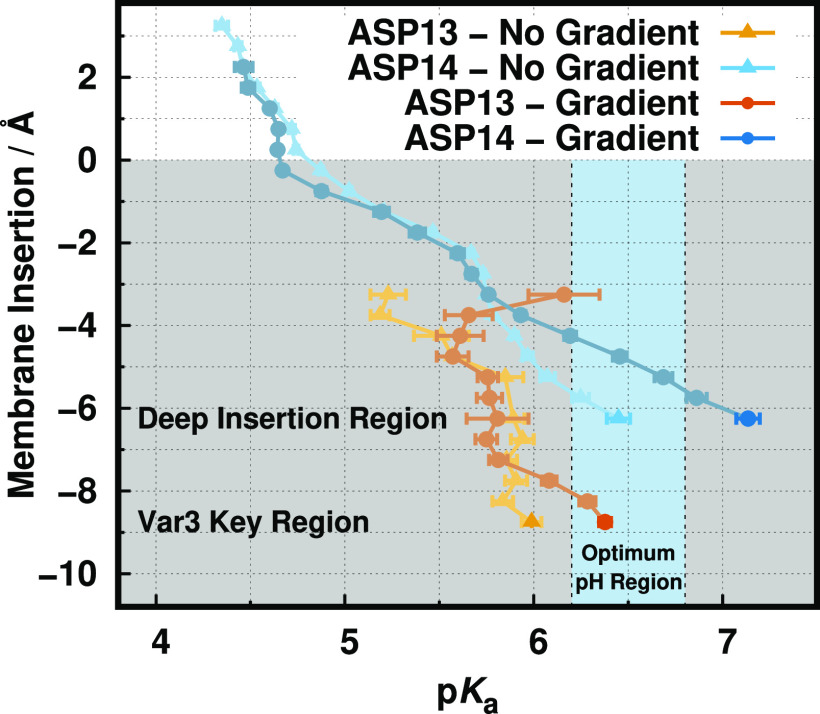
p*K*_a_ profiles of Asp-14 (*wt*) and Asp-13 (Var3) along the membrane normal for both simulation setups: gradient and no gradient. The white and gray-shaded regions correspond to the water phase and membrane interior, respectively. The light blue vertical stripe identifies the pH region ideal for TME selection. The Asp-14 no gradient data was adapted from ref (12).

The therapeutic performance of pHLIP peptides strongly depends on their ability to specifically penetrate the tumor membrane at acidic pH environments (6.2 to 6.8). Therefore, we need to calculate the p*K*_a_ profiles of the key aspartate residues (Asp-14 and Asp-13 for *wt* and Var3, respectively) and compare them with the available experimental data, which are either the peptide *in vivo* performance or the lysosome p*K*^ins^ values. The Asp-14 p*K*_a_ profiles are remarkably similar in both gradient setups (Figure 4), notably following the same expected trend where the p*K*_a_ shifts toward higher values, induced by desolvation effects.^36^ However, in the key deep membrane region (−5 to −6 Å), both profiles diverge to rather distinct p*K*_a_ values, 6.4 ± 0.1 and 7.1 ± 0.1 for nongradient and gradient, respectively. This indicates that different peptide–membrane configurations are being sampled at those residue insertions, confirming our initial assessment based on structural analysis. Moreover, these pools of configurations being sampled at the deeper insertion regions seem to be very homogeneous and/or well-mixed by the pHRE protocol in terms of both the conformations and the protonation states, which may explain the relatively small error bars in the p*K*_a_ profiles. Interestingly, the gradient setup insertion p*K*_a_ value falls outside the optimum pH region (light blue region), being in qualitative agreement with the experimental loss of performance of the *wt* peptide in cell experiments.^4^ Indeed, this performance can be correlated to the Asp-14 p*K*_a_ shift and the overall thermodynamic stability. The significant p*K*_a_ shift (+ ∼0.7 p*K* units) in the gradient setup indicates an increase in interactions with negatively charged groups, such as oxygen atoms from phosphates, fewer interactions with positively charged residues, such as the nearby Arg-11 or even choline groups, or a larger desolvation effect at similar membrane depths.

The gradient and nongradient Asp-13 (Var3) profiles follow the same p*K*_a_ increase, like the *wt* peptide (Figure 4). There is a clear shift in the region sampled by this peptide (Figure S6 of the Supporting Information), which results in an incomplete profile at more shallow (well-solvated) regions and a p*K*^ins^ region that is deeper (−8 to −9 Å) than in the *wt* peptide (−5 to −6 Å). The calculated p*K*_a_ values (6.0 ± 0.1 and 6.4 ± 0.1 for nongradient and gradient, respectively) at the deepest insertion region are in qualitative agreement with the experimental data since the gradient value falls within the TME optimum pH region.^76,77^ Nevertheless, the p*K*_a_ value estimated for liposome-like conditions is overestimated relative to the experimental p*K*^ins^ (5.0). The p*K*_a_ value of an unperturbed Asp residue at the water/membrane interface is usually >6.^7,36^ Therefore, we argue that to obtain such a lower value (5.0) an interaction with a positive residue is required, like Arg-9, which our model does not seem to fully capture. Such contribution would need to be selective for the nongradient setup, the one that seems to fall short of the experimental data.

**Figure 5:**
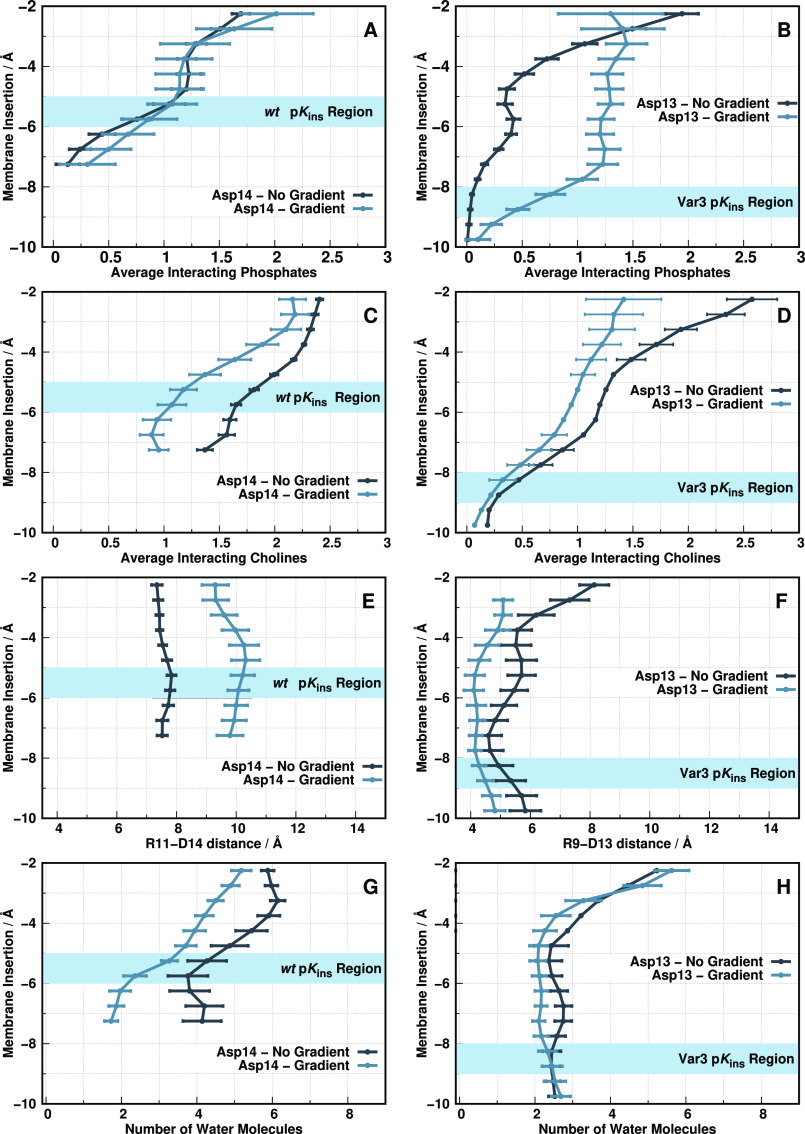
Electrostatic interactions of *wt* Asp-14 (A,C,E,G) and Var3 Asp-13 (B,D,F,H) with the surrounding molecular partners at pH 6.0 for both simulation setups. The interacting partners include the phosphate groups (A, B), choline groups (C, D), Arg-11/9 (E, F), and water molecules/desolvation (G, H). The phosphate/choline plots show the average number of interacting groups along the membrane normal. The arginine plots show the average interaction distance between Asp-14 and Arg-11 for *wt* and Asp-13 and Arg-9 for Var3. The desolvation plots show the average number of interacting water molecules with Asp-14 and Asp-13, respectively. The light blue horizontal stripe identifies the ideal insertion region for each peptide p*K*^ins^ estimation.

The p*K*_a_ values of the key Asp residues change in response to desolvation (membrane insertion) and to the neighboring electrostatic interactions. For these peptides, the most relevant players that establish strong interactions are the lipid phosphate and choline groups, the water molecules, and the nearby arginine residue (Figure 5). The observed *wt* Asp-14 p*K*_a_ shift in the gradient setup (+ ∼0.7 p*K* units) originates from a distinguishable electrostatic balance between this residue and the neighboring electrostatic groups. Since the number of interacting phosphate groups is the same in the two setups in the key membrane region (−5 to −6 Å) and Arg-9 remains relatively far away (Figure 5A,E), this effect seems to result from a slightly more pronounced desolvation effect coupled with a loss of interacting cholines relative to the nongradient setup (Figure 5C,G). The fewer choline groups within the first interaction shell may be the determinant factor, resulting in a less positive Asp-14 electrostatic vicinity in a pH gradient setup, while embedded in an apolar membrane environment, which typically favors the protonated state of carboxylic acids and, hence, higher proton binding affinities.

Concerning the Var3 behavior in both setups, the major distinguishing factor stems from more abundant phosphate contributions in the gradient setup at the p*K*^ins^ region (−8 to −9 Å) (Figure 5B). This effect can be somewhat counteracted by the slightly closer Arg-11 (Figure 5F), but the final p*K*_a_ shift in the gradient setup (+∼0.4 p*K* units) suggests otherwise. The remaining electrostatic contributions (Figure 5D,H) are indistinguishable between setups. As mentioned above, the main disagreement between our simulations and the experimental data available is in the nongradient setup, where the experimental p*K*^ins^ (5.0) is being overestimated by 1 p*K* unit in the calculations. To reproduce the experimental data, the nongradient simulations would require a larger contribution from a cationic partner (most likely Arg-11), which is not being correctly captured by our force field and/or our CpHMD-L simulations. If properly sampled, these interactions should reflect a stronger positive influence on Asp-13 that stabilizes its ionized form, shifting down the p*K*_a_ value, bringing it closer to the experimental p*K*^ins^ (5.0).

## 4 Conclusions

The p*K*_a_ values and protonation states reflect the electrostatic environment that a given titrating residue is sensing. The membrane (de)insertion process is triggered by key pH-dependent residues in the *wt* and Var3 peptides (Asp-14 and Asp-13, respectively), which interact with several local electrostatic partners. These include the lipid phosphate and choline groups, water molecules, and other neighboring residues, such as nearby arginines. The proton binding affinities of the aspartate residues are the product of a fine trade-off between all these interactions, and an incomplete model of these contributions may result in the wrong p*K*_a_ estimations and poor experimental correlation.

In this work, we extend the framework of our CpHMD-L methodology to include a membrane pH gradient setup. This novel protocol increases the level of realism when modeling cell membranes, particularly in the TME conditions where the exterior pH is significantly acidified, while the interior one is kept relatively stable (∼7.2–7.4). We showed that this pH-gradient setup impacts the configurational/protonation space of both peptides studied and helped us to rationalize the observable loss of performance of the *wt* sequence in tumor cell experiments. The Var3 peptide results also confirmed that the pH gradient setup is required to promote membrane insertion in tumor cells, which is in excellent agreement with the experiments. In sum, the pH gradient implementation was a pivotal step in bridging the *in silico* data to the *in vivo* experiments and identifying important electrostatic partners in pHLIP peptides. One of such partners, the “anchor” arginine residues, may have an important role as a direct modulator of the aspartate electrostatic vicinity and, possibly, the overall peptide thermodynamic stability. Although it is just a plausible hypothesis, it may warrant a future systematic study focused on the role of the arginine position in these peptides and how it modulates the surrounding peptide environment.

## Supporting Information Available

Membrane monolayer half-thickness values for the *wt*-pHLIP and Var3 peptides in both pH gradient and nongradient setups, local deformation profiles for *wt*-pHLIP and Var3 at pH 4.0, 5.0, and 7.0, average helicity content of *wt*-pHLIP and Var3 in both pH gradient and nongradient setups, and protonation profile and membrane insertion distribution of Asp-14 (*wt*) and Asp-13 (Var3) at different pH values in both pH gradient and nongradient setups (PDF)
