# Supplementary material for: Increasing the Realism of in Silico pHLIP Peptide Models with a Novel pH Gradient CpHMD Method
Source: J Chem Theory Comput. 2022 Oct 18;18(11):6472–81. doi: 10.1021/acs.jctc.2c00880 (PMC9775217; doi:10.1021/acs.jctc.2c00880)
Supplement: Supplementary file 1 — ct2c00880_si_001.pdf [file ct2c00880_si_001.pdf]

Supporting Information: Increase the realism of in silico pHLIP peptide models with a novel pH gradient CpHMD method

Tomás F. D. Silva, Diogo Vila-Viçosa, and Miguel Machuqueiro*

BioISI – Instituto de Biosistemas e Ciências Integrativas, Faculdade de Ciências, Universidade de Lisboa, 1749-016, Lisboa, Portugal

E-mail: machuque@ciencias.ulisboa.pt

Phone: +351-21-7500112

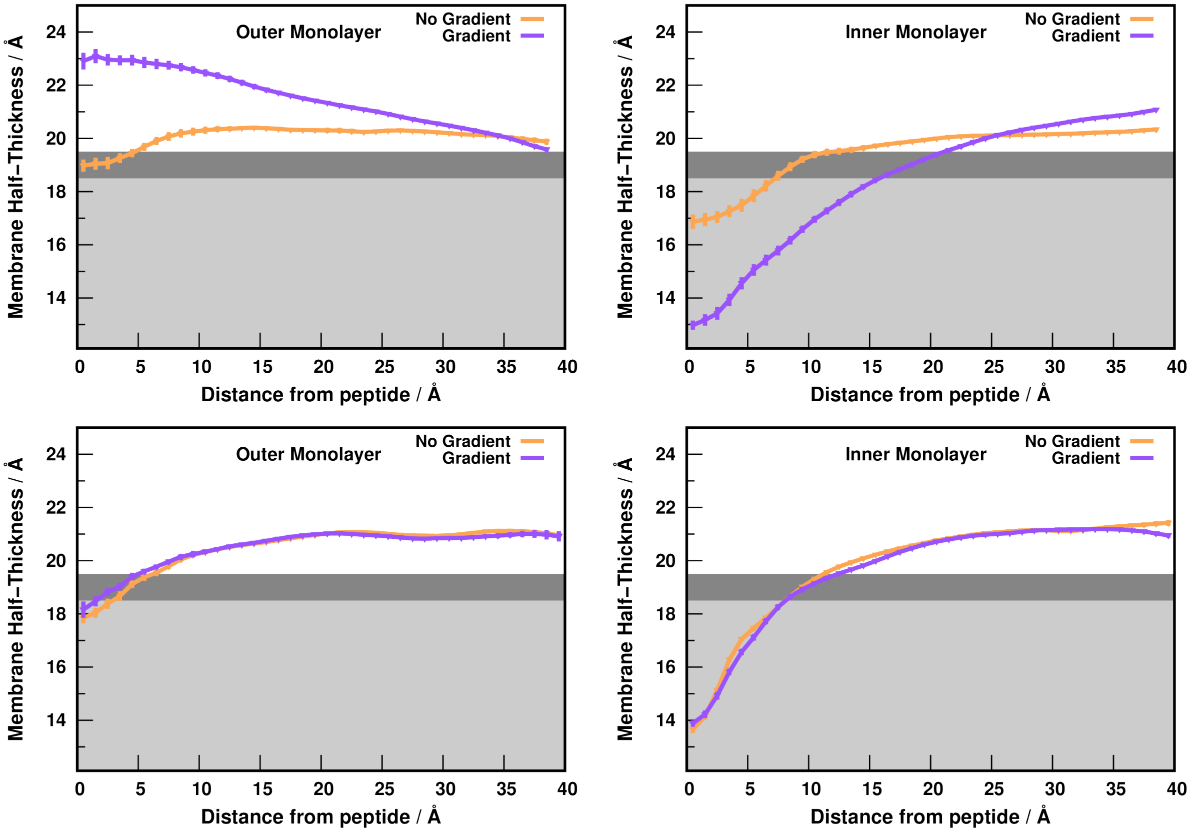

Figure S1: Outer (left) and inner (right) monolayer half-thickness values in the xy plane along the *wt* (top) and Var3 (bottom) peptides distance. The representations show the membrane deformations induced by the peptides, in both setup conditions at pH 6.0, up to the 'bulk' lipids (15–40 Å away). The error bars were calculated from the SEM using all replicates every 0.5 Å and they were plotted every 1 Å for visual clarity. The grey shaded region is the membrane interior, while the dark grey region corresponds to the experimental range of fluid POPC (∼19 Å for each monolayer).[S1]

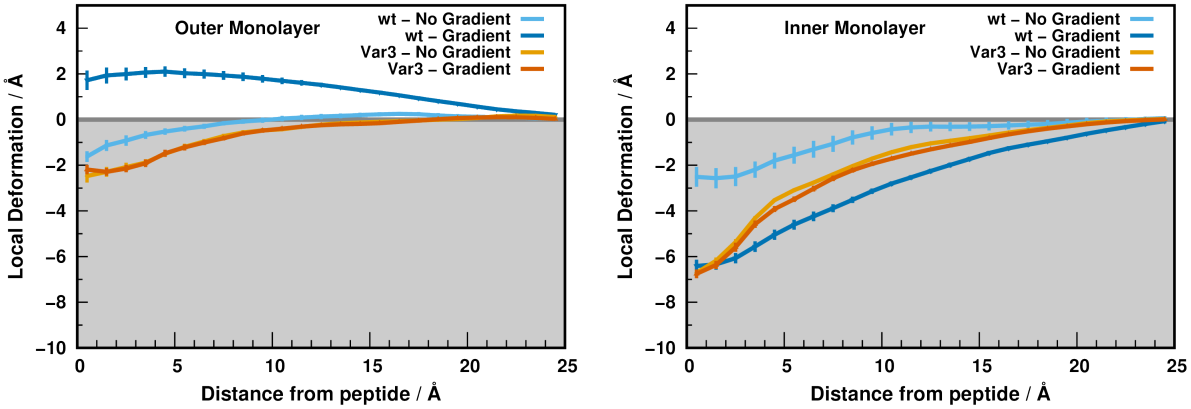

Figure S2: Outer (left) and inner (right) local monolayer deformations induced by the *wt* (blue/cyan) and Var3 (orange/brown) peptides, in both setup conditions at pH 4.0. These deformations values are calculated from the half thickness values calculated along the *xy* plane distance to the peptides. The maximum distance shown (∼ 25 Å) corresponds to the unperturbed 'bulk' lipids. The error bars were calculated every 1 Å, using all replicates, with a SEM method and they were plotted every 1 Å for visual clarity. The grey-shaded region corresponds to the membrane interior.

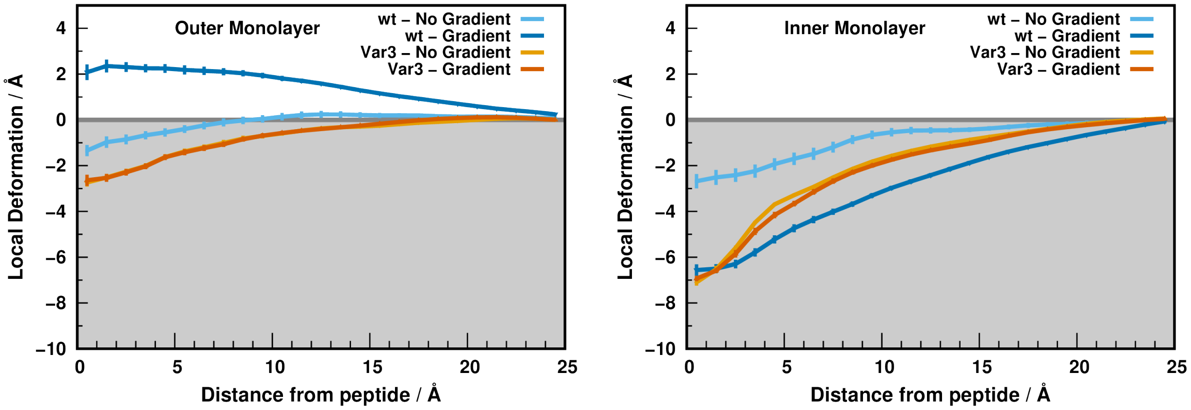

Figure S3: Outer (left) and inner (right) local monolayer deformations induced by the *wt* (blue/cyan) and Var3 (orange/brown) peptides, in both setup conditions at pH 5.0. These deformations values are calculated from the half thickness values calculated along the *xy* plane distance to the peptides. The maximum distance shown (∼ 25 Å) corresponds to the unperturbed 'bulk' lipids. The error bars were calculated every 1 Å, using all replicates, with a SEM method and they were plotted every 1 Å for visual clarity. The grey-shaded region corresponds to the membrane interior

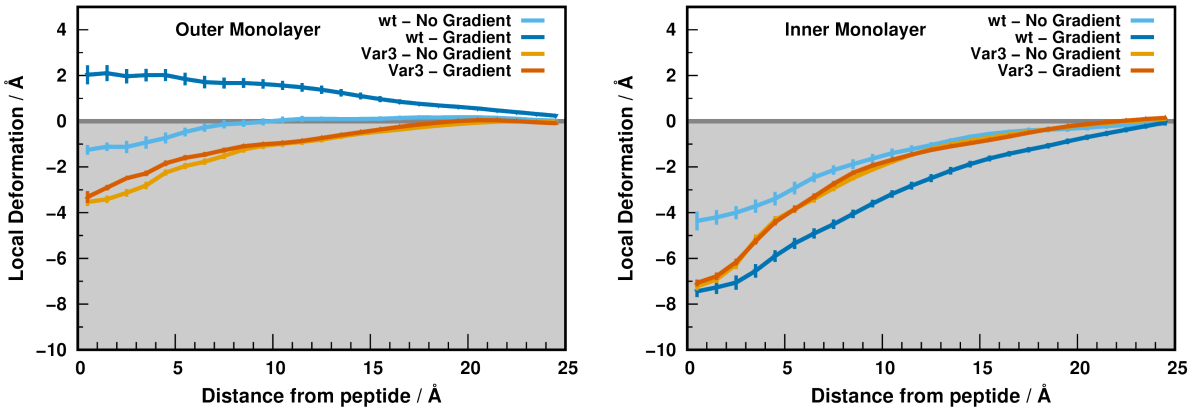

Figure S4: Outer (left) and inner (right) local monolayer deformations induced by the *wt* (blue/cyan) and Var3 (orange/brown) peptides, in both setup conditions at pH 7.0. These deformations values are calculated from the half thickness values calculated along the xy plane distance to the peptides. The maximum distance shown ($\sim$ 25 Å) corresponds to the unperturbed 'bulk' lipids. The error bars were calculated every 1 Å, using all replicates, with a SEM method and they were plotted every 1 Å for visual clarity. The grey-shaded region corresponds to the membrane interior

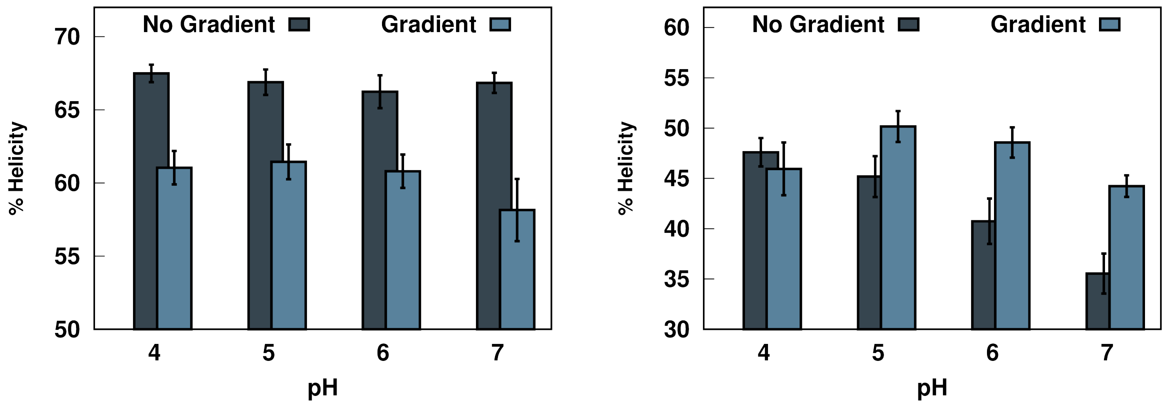

Figure S5: Percentage of α-helix content of *wt* (left) and Var3 (right) peptides in both gradient and non-gradient pH setups, at all pH values. Note that, in the gradient setup, pH refers to the pH_{out}, since pH_{in} is fixed at 7.2. The average percentage of helicity was calculated using the equilibrated segment of all replicates. The error values were calculated with a standard error of the mean (SEM).

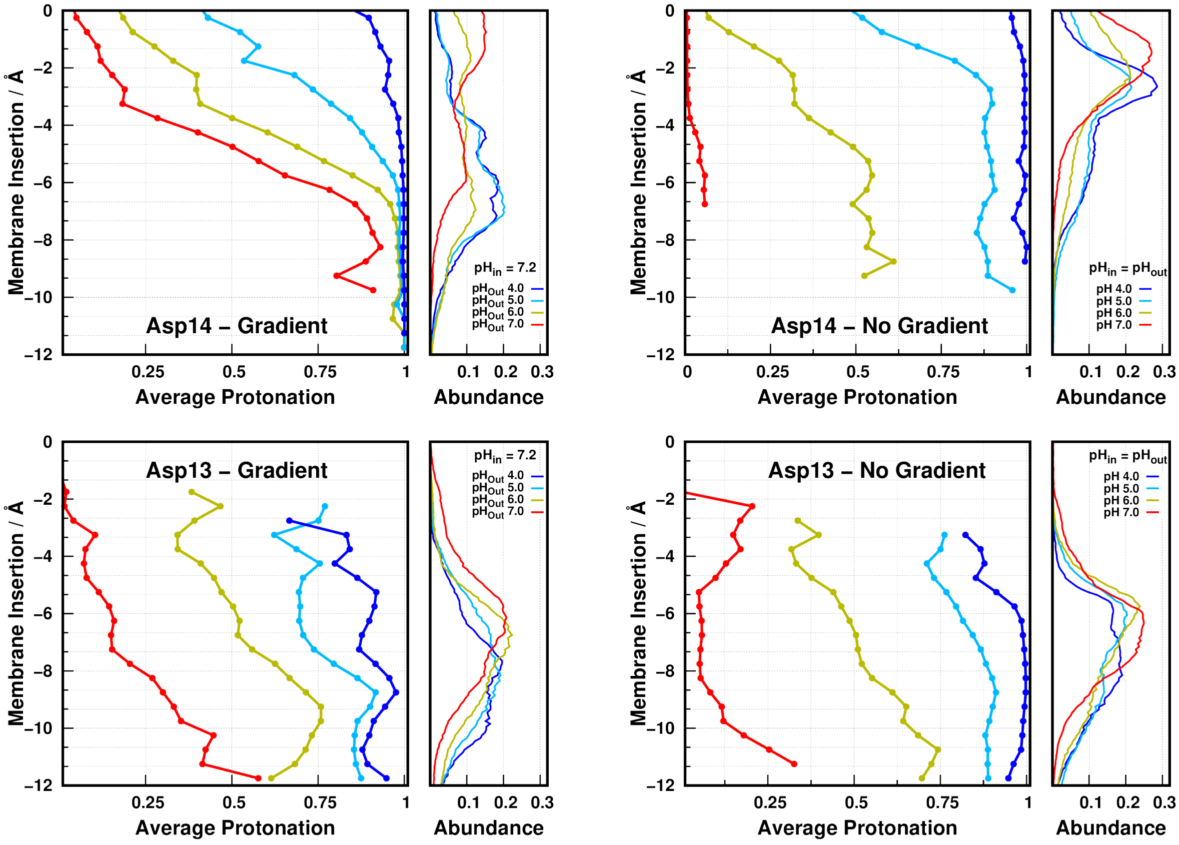

Figure S6: Protonation profiles of Asp14 (*wt*; top) and Asp13 (Var3; bottom) in a Gradient (left) and no Gradient (right) setup. The profiles indicate the average protonation of the Asp residue, at each pH value, for each insertion slice (left sub-plots) and the histogram distribution of residue insertion in the membrane for each pH value (right sub-plots). The protonation errors bars were omitted for clarity and the average error size was ∼0.1.

References

(S1) Kučerka, N.; Nieh, M.-.; Katsaras, J. Fluid phase lipid areas and bilayer thicknesses of commonly used phosphatidylcholines as a function of temperature. *Biochem. Biophys. Acta, Biomembr.* **2011**, *1808*, 2761–2771.
